# Is there a link between genetic defects in the complement cascade and *Porphyromonas gingivalis* in Alzheimer’s disease?

**DOI:** 10.1080/20002297.2019.1676486

**Published:** 2019-10-25

**Authors:** Ingar Olsen, Sim K Singhrao

**Affiliations:** a Department of Oral Biology, Faculty of Dentistry, University of Oslo, Oslo, Norway; b Dementia and Neurodegenerative Diseases Research Group, Faculty of Clinical and Biomedical Sciences, School of Dentistry, University of Central Lancashire, Preston, UK

**Keywords:** GWAS, periodontitis, *P. gingivalis*, immune subversion, complement, dysbiosis

## Abstract

Defects, as determined by Genome-Wide Association Studies (GWAS), in the complement cascade of innate immunity have been suggested to play a key role in Alzheimer’s disease (AD). These defective genes encode sub-component 1s (C1s), complement receptor 1, complement component 9, and clusterin, a fluid-phase regulatory protein. A dysregulated complement cascade has been shown to relate to cell activation, defective complement mediated clearance and possible cognitive decline in AD patients. *Porphyromonas gingivalis*, a putative keystone pathogen of periodontal disease, has been reported to be associated with human AD. The inflammatory burden following experimental oral infection in mice and putative entry of this bacterium into the brain appears to drive the formation of amyloid-beta plaques and neurofibrillary tangles with loss of cognition. *P. gingivalis* is a master of immune subversion in this inflammatory cascade and may establish microbial dysbiosis where it is located. Here we discuss if *P. gingivalis* may enhance the detrimental effects of the defective GWAS complement cascade protein genes.

Alzheimer’s disease (AD) is a neurodegenerative disease and the most common form of dementia. It differs from other forms of dementia by the presence of two hallmark proteins, amyloid-beta (Aβ) plaques and hyperphosphorylated tau bound to neurofibrillary tangles (NFTs). The cause of AD remains largely undefined. It is widely accepted that this complex neurological condition can co-exist with other complex diseases such as atherosclerosis and cerebrovascular/ischemic stroke [1–5]. The link with complex diseases is the *apolipoprotein E* gene allele 4 (*APOE є4*) inheritance [6–8]. The consequence of the *APOE є4* inheritance is defective complement activity [9,10] because this isoform resists the innate immune cascade checkpoint control at C1q which is a subcomponent of the complement C1 complex [8]. Sustained complement activation is a potent driver of inflammation in the body including the brain [11–15]. Moreover, the pathological lesions (Aβ plaques and NFTs), microbial pathogens, and physical injury can activate this innate immune cascade extracellularly as depicted by Aβ and/or intracellularly as per NFT bearing neurons [11–15]. This effectively makes it impossible to disregard an unresolved complement pathway activity in AD.

Over the years several pathogens of bacterial, viral and fungal origin have been shown to be associated with AD brains [16]. However, the etiologic role of these microbes in AD pathogenesis is still in question. Recent studies have proposed that the putative keystone periodontal pathogen *Porphyromonas gingivalis* can be a risk factor that contributes to AD development in some individuals [17]. Periodontitis is a chronic inflammatory disease affecting the tooth supporting tissues, caused by polymicrobial dysbiosis [18,19]. It has been proposed that imbalance in complement activity may influence dysbiosis of host microbiomes [20]. Pathogens adopt and adapt to survival and utilization of longstanding inflammatory environments as demonstrated by the presence of *P. gingivalis* in the subgingival crevice (as commensal and pathogen) and at distant sites (heart, placenta, and perhaps brain) with inflammatory components for the development of systemic diseases [21].

Aβ plaques and NFTs have been detected in brains of mice with the sporadic form of AD after infection with *P. gingivalis* [22]. Dominy et al. [17] showed that the enzymes gingipains produced by *P. gingivalis* can degrade the Tau protein, which is involved in NFT formation in AD. In mouse brains, all these lesions, purported entry of *P. gingivalis*, complement activation and *APOE* gene knock-out can accompany intracerebral inflammation [22,23]. The recognized innate immune subversion caused by *P. gingivalis*, the antimicrobial protection hypothesis for lesions [24], and genetic polymorphisms in some complement genes [25–28] have relevance towards a basis for complement imbalance in AD. Lamont et al. [29] proposed that longstanding inflammatory conditions of the brain, typically AD, are related to growing old. During the lifespan of man there are changes both in the architecture such as increased permeability in the blood-brain barrier (BBB) of the hippocampus [30], and functioning of the immune system (immunosenescence) [31]. The term immunosenescence refers to decline in fidelity and efficiency with age, resulting in an increased susceptibility to infectious diseases and pathological conditions relating to inflammation (e.g. cardiovascular disease and AD) or autoreactivity (e.g. rheumatoid arthritis) as described by Caruso et al. [32].

## The complement system

**Figure 1.**
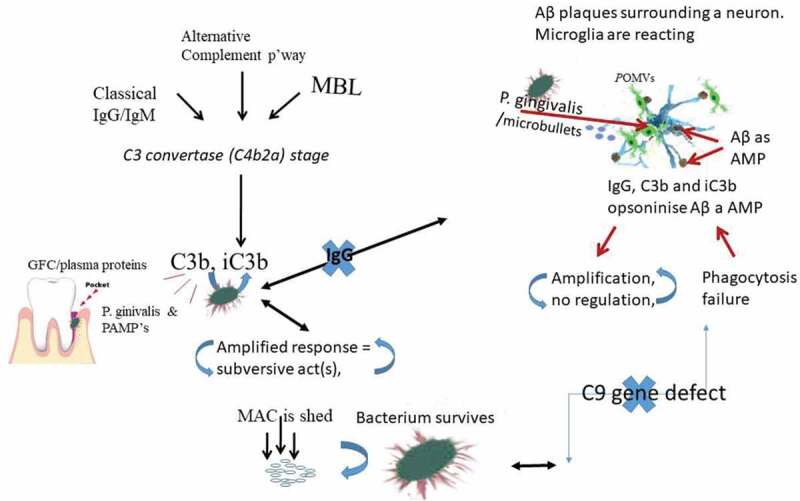
Illustration showing the effects of *P. gingivalis* oral infection and its local subversive effect on degradation of opsonins with IgG, C1q, iC3b and MAC to evade complement mediated death and at the same time amplify inflammation. In the brain, a nerve cell infected by *P. gingivalis* itself or internalization of outer membrane vesicles (microbullets) initiate microglial surveillance. This results in an inflammatory activity when the host cell encounters Aβ (in its capacity as an AMP) opsonized by IgG, C3b and iC3b opsonins in the paths of the neuronal processes. Due to polymorphic defects in the complement regulating proteins, and the inability of microglia to clear Aβ, inflammation is thought to be amplified and sustained

**Figure 2.**
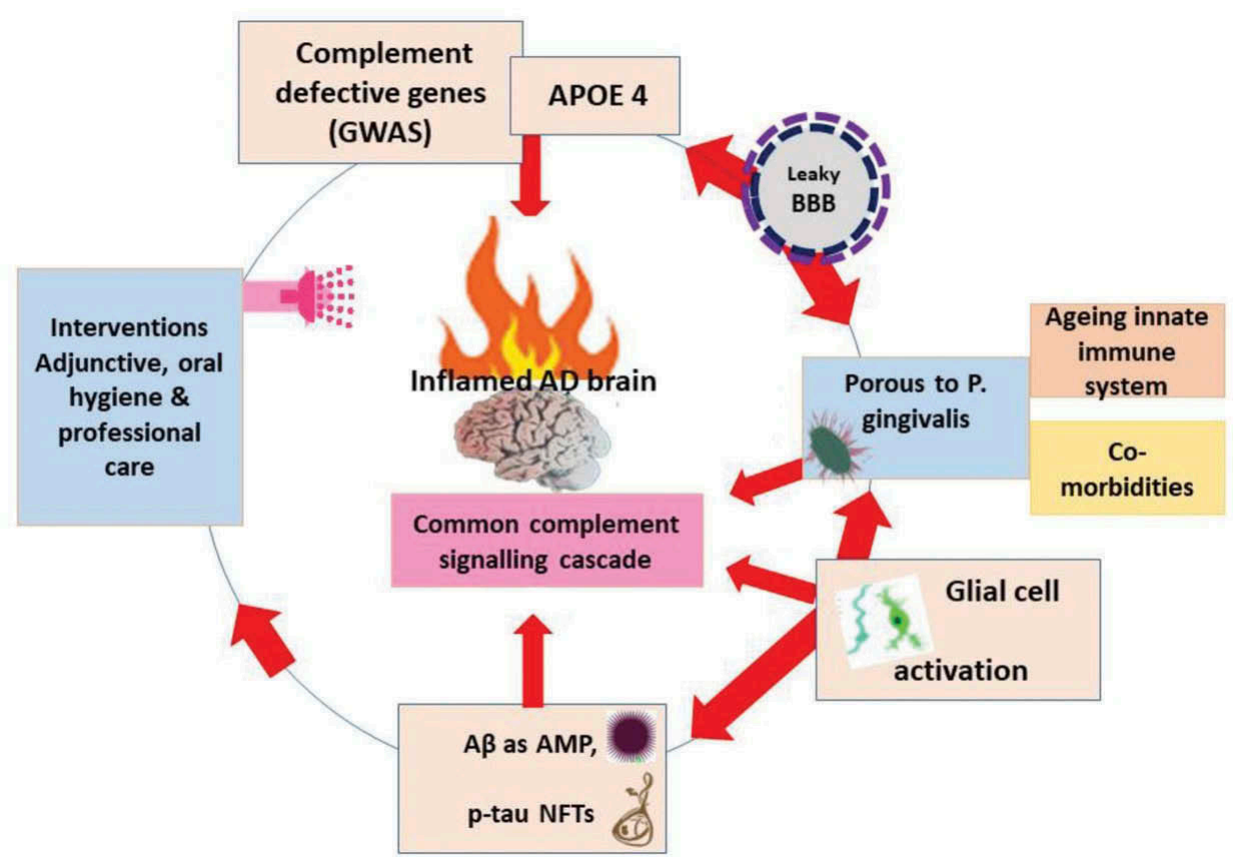
A ‘frustrated innate immune system’ in the inflamed Alzheimer’s disease brain. This contribution is from multiple sources including the polymorphic complement component genes [26–28], the APOE variant [8], blood-brain barrier defects [30], pathogen entry, and Aβ as a defense peptide released in response to infections [24]. All these contribute to complement activity, cell activation, defective phagocytosis and chronic inflammation [15]. There would be clinical value in inhibiting all three main pathways of complement at the C3 stage

The complement system is comprised of more than 50 proteins, including the component proteins C1-C9, which are part of the innate immune system. There are regulatory proteins that serve to inhibit the complement cascade at various points [29]. The effector molecules (opsonins) illicit ongoing damage and initiate signaling cross-talk. Examples of membrane bound regulatory proteins include membrane cofactor protein (MCP or CD46), decay-accelerating factor (DAF or CD55), complement receptor 1 (CR1 or CD35), and CD59. The soluble or fluid phase regulators, which form the focus of this review, are C4 binding protein (C4bp) and clusterin. Complement can be activated through the classical, alternative or lectin pathways [33]. An antibody bound to antigen or a solid surface can activate the classical pathway. Spontaneous hydrolysis of the complement protein C3 or binding of C3b to microbes activates the alternative pathway through the feedback loop, while mannose moieties on bacteria activate the lectin pathway [33–35]. All these pathways merge at the C3 convertase (C4b2a) stage, which causes hydrolysis of C3 into C3a and C3b fragments [33], see Figures 1 and 2. While C3a is a potent anaphylatoxin that regulates immune responses such as inflammation in the fluid phase, C3b opsonizes target cells and promotes activation of the terminal complement pathway, which ends with the assembly of the membrane attack complex (MAC) on target cells destined for killing [36]. All nucleated human cells can limit the lytic effect of the activated complement by expressing complement regulatory proteins [37]. However, gene polymorphisms may have major effects on the function of specific gene defects. Hence with polymorphic complement cascade genes identified in AD, we know little about their contribution to the overall effect on disease pathogenesis.

## Genome-wide association studies link defects in the complement cascade with Alzheimer’s disease

Genome-Wide Association Studies (GWAS) [25–28] reported the four defective genes that potentially link to AD progression: 1) complement sub-component 1s (C1s); 2) complement receptor 1; 3) complement component 9; and 4) clusterin, a fluid-phase regulatory protein. This is of concern because the brain, unlike other organs, is devoid of a traditional lymphatic vasculature system, meaning that an efficient complement cascade is critical for clearance of damaged cerebral tissue debris. Consequently, defective complement genes scattered within the early, middle and late stages of this cascade may be responsible for disabling the phagocytic activity of local microglia, resulting in inefficient removal of waste proteins such as Aβ and possibly ‘ghost’ NFTs (tangles without cell surface membrane of the neuron) as typically seen in AD brains. An added complication of the AD brain is its association with microbes.

## Complement proteins of polymorphic genes relevant to Alzheimer's disease

### C1s

C1s complexes with two molecules, C1r and C1q, and form C1 as the first component of the classical complement activation. C1 is a serine esterase that activates C4 and C2 thereby driving the classical pathway of complement activation [38]. C1 is not stable as it dissociates rapidly by the activity of the fluid phase regulator C1 inhibitor [39]. Interestingly, the virulence associated gene 8 (*Vag8*) in *Bordetella pertussis* is a complement evasion molecule that mediates its effects by binding to the complement regulator C1 inhibitor (C1-INH), which is a fluid phase serine protease [40]. The absence of functional C1s (defected gene) suggests that C1 cannot be activated in the context of its ability to initiate the classical complement cascade [41]. In this scenario, the resident microglial cells that express the phagocytic C1qR receptor [42] would fail in their function. However, if the C1s subcomponent is seen as an inactive protein, this could represent a pool of ‘inactivated’ C1. ‘Inactivated’ C1 can complex with C1r and C1q and activate the classical complement pathway [41]. Literature supports incomplete complement activation in AD brains [11–15]. This suggests that ‘inactivated’ C1 eventually binds to other ‘activators’ (Aβ, NFTs, microbial pathogens, physical injury) which propagate the incomplete complement pathway activity by cleaving the next component in the cascade in demented brains.

### Cr1

The GWAS support a *CR1* gene defect in AD. This finding appears reasonable as AD patients have reduced resistance to infection by bacteria and viruses [16]. In the context of bacterial infections for the pathogenesis of AD, prominence is being given to *P. gingivalis* [17,23] that reaches the brain via the haematogenous route and *Chlamydia*-associated infections that are able to reach the brain via infected monocytes following increased BBB permeability [43]. The herpes simplex virus (HSV) type 1 [44] infections are endemic in the host and they become re-activated in some individuals leading to AD. All the above listed infections associate with the Aβ hallmark lesion of AD [22,45,46], and both *P. gingivalis* infection and the HSV type I infection lead to cognitive deficit in mice [22,46].

CR1, also known as the C3b/C4b receptor, is a transmembrane glycoprotein that functions to inhibit activation of the C3/C5 convertase stage of the three converging activation pathways. Hence the location of CR1 in the complement cascade is pivotal to all subsequent effector pathways. CR1 helps to regulate activation of the complement cascade and promotes phagocytosis of cellular debris, as well as Aβ plaques, and adherence of immune complexes to erythrocytes. It has been demonstrated that the AD brain is generally deficient in CR1 [37]. Notably, it has been reported that *P. gingivalis* infection mediates immune subversion in relation to CR1 [47]. Such observations reinforce regions of genetic weaknesses (as per *CR1* gene defect, see [26]) that are also exploited in this case by *P. gingivalis*, albeit in a fully functional complement system.

### C9

Complement component protein C9 is part of MAC, and its insertion into cell surface membranes induces pores to lyse target cells. Other than the GWAS, little is known about the deleterious effects of the defective *C9* gene in relation to AD pathogenesis, or indeed in other complement deficiency related conditions. The earliest reports linking complement to Aβ plaques suggest that the activated complement cascade does not proceed to C9/MAC formation [48,49]. Whether or not such an observation points to an underlying genetic defect in the *C9* gene or incomplete activation of the complement cascade in presence of active genes remains to be clarified. However, if the defective *C9* gene has lost its function, this may be one factor that can influence dysbiosis of the host’s oral/gut microbiomes as reported for AD [50]. One possibility is that the functional loss of complement activity (unable to kill the pathogen) would support the spread of microbes such as *P. gingivalis* in the body via increased permeability of the BBB in the elderly and the AD brains. Having established *P. gingivalis* colonization, the bacteria would dampen the proinflammatory activity of C5a by citrullination (discussed below). Thus, there remains a potential for a microbial component of AD brains that could promote rampant complement activation (due to gene deregulation) and resulting excessive inflammation.

### Clusterin

The polymorphism in the clusterin gene has a more convincing role in the pathogenesis of AD, relating to subtype (mild cognitive impairment and dementia), and the rate of progression [51–53]. It is one of the complement cascade regulatory plasma proteins that significantly increases during AD as compared with non-AD controls [54]. Clusterin also stimulates expression and secretion of various chemotactic cytokines, including tumor necrosis factor-alpha (TNF-α), which plays a critical role in promoting macrophage chemotaxis via the phosphoinositide 3-kinase/protein kinase B1 (Pi3K/Akt), mitogen-activated protein kinase/extracellular signal-regulated kinase (ERK) and c-Jun N-terminal kinase (JNK) pathways [55]. Pathogen-driven signaling pathways with kinases that phosphorylate proteins may also be involved in abnormal phosphorylation of Tau proteins, which are the major constituents of NFTs in AD. This possibility was illustrated by Ilievski et al. [22] who demonstrated ser396 phosphorylation following *P. gingivalis* oral infection in mice. Alternatively, gingipains can digest the normal Tau protein into fragments that may be toxic to neurons [17]. Further research should clarify the concomitant role of *P. gingivalis* and polymorphic complement genes in AD pathogenesis.

## The role of Aβ plaques and NFTs in the classical complement pathway activation in Alzheimer's disease

The inflammatory component of AD was recognized through the classical complement pathway activation and receptors for specific (C3a, C5a) opsonins [11,12,56,57]. Based on these early data, the fibrillary insoluble Aβ plaques were suggested to act as extracellular triggers of complement activation [58–61]. NFTs are intracellular triggers of complement activation via the classical pathway [62]. Building on the Dominy et al. [17] observation that gingipains degrade Tau protein could reveal new triggers of intracellular complement activity in AD brains.

## Apolipoprotein E-C1q complexes as inhibitors of the activated classical complement pathway

It is becoming clear that the *APOE є4* susceptibility gene may be linked to deregulating C1q to keep the classical complement pathway activated [8]. This causes a dysregulated innate immune inflammatory response via cytokine liberation by activated monocytes/macrophages/microglia [63]. In the brain, the oxidized lipids also accumulate at the periphery of Aβ plaques [8], which leads to yet more activation of complement activity. The *APOE є4* susceptibility gene is also linked with environmental risk factors, including the host’s dysbiotic oral microbiome [64]. The sustained inflamed environment of the brain could act as an intrinsic environmental factor that supports dysbiosis.

## Synaptic loss: a potential consequence of activated complement cascade

The phagocytic role in the brain is well-recognized as an arm of the complement cascade and generally regarded as being beneficial to the host. However, in AD brains activated complement CR1 helps to regulate and promote phagocytosis, in microglia, of the cellular debris. With the CR1 activity being suppressed (via the deregulated *CR1* gene and immune evasion strategies of *P. gingivalis*) this would suggest accumulation of abnormal proteins. Although this is the case in AD, an additional outcome appears to be the excessive loss of synapses. This is supported by *in vivo* studies, where the classical complement pathway was activated via (oligomeric) Aβ and led to excessive pruning of synapses by microglia [65].

## Concept of cognitive deterioration in Alzheimer's disease

Cognitive deterioration (difficulties in decision making and deteriorating mental function with changes in mood and behaviors) is an essential component of the clinical picture of AD. Exactly what causes functional loss in AD remains unknown. However, the original synaptic loss theory [66,67] is still considered valid, as it continues to correlate with deteriorating memory. A question arises as if the mechanism of overt synaptic loss relates to an overactive complement cascade [65,68]. Observational studies demonstrate that some very elderly subjects bypass AD, whilst harboring equivalent numbers of Aβ plaques and NFTs in their brains. This suggests that these lesions, *per se*, do not necessarily cause functional deficits. Such individuals have been termed as having a ‘cognitive reserve’ [69,70]. Another group of elderly patients’ brains have shown extensive numbers of Aβ plaques and NFTs without the individual receiving a diagnosis of clinical AD. These individuals have been referred to as having ‘resilient’ brains [71]. The difference between those with cognitive reserve and resilient brains, over individuals with AD, is the absence of intracerebral inflammation [71]. This observation emphasizes the role of chronic inflammation, in some individuals, for the functional loss.

## Microbial component in Alzheimer's disease pathophysiology

The studies carried out by Vasek et al. [68] supporting the role of complement activity via an initial infection causing overt pruning of synapses and giving rise to clinical symptoms, can be explained through classical plaques. Research has linked Aβ to a broad-spectrum antimicrobial peptide (AMP) [72–74]. If Aβ deposition represents the host’s response to a previous infection, then its role as an AMP is consistent with triggering complement activation [24,73]. This forms a common link with the antimicrobial protection hypothesis [23], whereby the modality of Aβ’s pathophysiology is shifted towards a dysregulated innate immune response, and indirectly, with the microbial infection hypothesis and the amyloid cascade hypothesis [75]. The only difference is that that the amyloid hypothesis maintains that Aβ is toxic, whilst the antimicrobial protection hypothesis suggests AD pathology develops from a pattern of innate immune responses mounted by an immune challenge.

*P. gingivalis* is an oral pathogen that has been used to develop models for periodontal infection and AD in mice [22,23]. Most interestingly, the periodontal infection model of Ilievski et al. [22] has demonstrated Aβ and NFTs in mouse brains. Therefore, by example of *in vivo* bacterial infections, *P. gingivalis* gives rise to Aβ in the brain produced by the host with implication for pathogen entrapment and killing. This confirms that *P. gingivalis* can initiate Aβ and NFT formation and that this, over time, will contribute to the overall burden of AD lesions (Figure 2). In addition, *P. gingivalis* activates complement in the absence of Aβ in the brain [23]. Complement activation following bacterial entry into the brain is to be expected, but this observation may also explain memory impairment possibly through intercommunication with toll-like receptor (TLR) activation, lipopolysaccharide (LPS) (a TLR4 agonist) and complement activation [49,76–78]. Collectively, they may also cause loss of synapses [65,68] if CR1 functionality is suppressed by its polymorphism or via immune evasion strategies of bacteria like *P. gingivalis*.

## *P. gingivalis* and its complement subversion

*P. gingivalis* has been shown to be a major manipulator of the immune system [79–84] and is considered a keystone pathogen in ‘chronic’ periodontitis [85]. Furthermore, periodontitis has a clear relationship with late onset AD, which is the most common form of AD [86–90]. *P. gingivalis* LPS and gingipains can suppress the deposition of opsonins (IgG, C3b, C5b-9) on the bacterial cell surface [81]. Blocking C3, on which all complement pathways converge, would allow for infection to take hold. Such an action might be detrimental for older peoples’ oral and mental health because *P. gingivalis* can remodel the oral microbiota into a dysbiotic state by exploiting complement [42,81,84]. Subversive mechanisms are important for the collective virulence of microbial communities where *P. gingivalis* exists. However, *P. gingivalis* is not the only microorganism present in its primary location, subgingival plaque, and not even in the multitude of bacteria detected in brains from AD cases [91]. Therefore, although *P. gingivalis* may be important in AD, its role has yet to be defined and proved.

## Gingipains as players in immune subversion

Gingipains are virulence factors of key importance to the immune subversion activity of *P. gingivalis*. There are two main types of cysteine proteases [92] encoded by three different genes (*rgpA, rgpB* and *kgp*). Of these the lysine specific gingipains is the product of *kgp* and the arginine specific gingipains *rgpA* and *rgpB*. These proteases can cleave the complement components C1-C5, prevent deposition of C3b on the bacterial surface and capture the C4b binding protein [93–97]. By binding to the complement regulator C4bp on the bacterial surface, *P. gingivalis* prevents assembly of the membrane attack complex and acquires the ability to regulate C3 convertase [95]. Thus, gingipains do not only destroy complement through proteolytic degradation, they also inhibit complement activation by binding to the complement inhibitor C4bp [95].

If gingipains are involved in AD, they would likely enhance the effect of polymorphic complement gene defects, allowing for a local infection. Recruitment of additional bacteria that are resistant to the bactericidal activity of complement is also feasible [94]. Besides, it is possible that gingipains, together with defective complement component genes, aggravate and sustain AD through ineffective clearance of cellular debris, which in turn, aids the accumulation of Aβ and NFTs. Tau protein that is associated with NFTs in AD brains is reported to be a substrate for gingipains [17]. Whether this is a strategy of *P. gingivalis* to keep complement activated or is independent of complement requires further research.

## *P. gingivalis* and citrullination in Alzheimer's disease

*P. gingivalis* can also reduce the antibacterial and proinflammatory activity of C5a by deiminating its C-terminal arginine [98]. Post-translational enzymatic modification of arginine residues in proteins formed as part of the complement cascade are some of the subversive physiological processes demonstrated by *P. gingivalis*. This offers a plausible and exclusive link to disabling complement C5a enzymatic conversion of arginine to citrulline. Protein citrullination causes deregulation of the host’s inflammatory signaling network by altering the spatial arrangement of the original 3D-structure and function of immune proteins [99]. It is likely that degradation of complement proteins allows colonization and proliferation of bacteria possessing higher sensitivity towards complement mediated killing than found in *P. gingivalis* itself [82]. Accordingly, *P. gingivalis* may support survival of an inflammophilic biofilm community by helping bystander bacteria in the brain to evade complement-mediated killing. The concept of the brain possessing its own microbiome has not been fully explored and future studies will undoubtedly reveal whether alternative mechanisms exist for complement activation not proceeding to C9/MAC formation in AD [48,49].

## *P. gingivalis* and its possible subversion of CR1 and clusterin

The presence of CR1 on peripheral blood cells, especially erythrocytes and macrophages, is abundant and suggestive of an important and significant role of CR1 in AD. For example, as a receptor for the components C3b and C4b, CR1 helps to regulate activation of the complement cascade and promotes phagocytosis of cellular debris, as well as Aβ and adherence of immune complexes to erythrocytes.

Clusterin is a plasma protein that may play an important role in regulating C5b7-8 stages of the terminal complement complex pathway, and in the subsequent pathogenesis of AD. The blood plasma analysis of APP/PS1 AD transgenic mice demonstrated greater concentration of clusterin, and an age-dependent upregulation in the brain, and its co-localization with Aβ plaques [48,51,52]. Clusterin also stimulates expression and secretion of various chemotactic cytokines, including TNF-α, which plays a critical role in promoting macrophage chemotaxis, via the Pi3K/Akt, ERK and JNK pathways [55].

Data from GWAS suggest an involvement of CR1 and clusterin gene defects in AD [25–28]. Since *P. gingivalis* has the capacity to affect CR1 and clusterin, this strengthens the possible pathogenic role of this bacterium in AD, at least through increased immune subversive activity. For example, outside the brain, *P. gingivalis* was found to fix C3 and readily adhere to erythrocytes via CR1, and this led to a rapid degradation of C3 into iC3b, and presumably, C3dg on the erythrocyte cell surface [100].

## *P. gingivalis* and its possible subversion of C9

C9 is the ninth complement component protein, which is also a part of MAC. Its insertion into cell surface membranes induces pores, causing lysis. *P. gingivalis* gingipains (Kgp, RgpA, and RgpB) degrade the central complement component C3. This prevents deposition of both C3b opsonin and MAC on *P. gingivalis* cells, by which the bacterium protects itself against complement [101]. It is known that the complement cascade does proceed to MAC formation in periodontitis, and this is due to the membrane bound regulator CD59 being partially effective. This allows for degradation of collagens and heme, which form essential nutrients for the bacterium. Generalized gene defects are conducive to this exploitation, as reported by Kapferer-Seebacher et al. [102] for the effects of C1S gene mutation in periodontitis in patients with Ehlers-Danlos syndrome. Such findings support sustained inflammation in periodontitis and AD brains, and the GWAS finding of the defective *C9* gene causing deficiency in overall C9 protein synthesis, might primarily affect the brain.

To date, there is only one report that tested complement activation in mouse brains. It confirmed entry of *P. gingivalis* [23] and demonstrated MAC on some neurons. Although the difference from sham treated animals was not statistically significant, the data suggested that *P. gingivalis* may have the capacity to suppress the activity of C9 and impair MAC assembly via immune subversion.

## *P. gingivalis* and epigenetic modifications

In the stimulation and maintenance of inflammation epigenetic pathways have received special attention because of their upstream regulations. Epigenetic modifications lead to chemical changes in DNA and associated proteins which cause remodeling of the chromatin and activation or inactivation of gene transcription. These changes can contribute to development and maintenance of cancer, autoimmune and inflammatory diseases, including periodontitis [103,104]. Interestingly, knowledge of the modification of epigenetic mechanisms may provide insight into key regulatory pathways of genes involved in the maintenance of chronic inflammation. Thus, the role of DNA and histone modifications, which are major epigenetic regulations, have been described in periodontitis where gene expression can be affected by DNA methylation [105]. It has also been demonstrated that chronic inflammation in periodontitis may be linked with aberrant DNA methylation in the gingival tissues [106,107]. In AD, epigenetic mechanisms have been found to be dysregulated during disease progression, already in its early stages [108]. Furthermore, recent methylome-wide association studies (MWAS) in humans have supported the concept that aberrant DNA methylation is associated with AD [109]. Whilst increased methylation in the gene promoter region is related to reduction in gene expression, hypomethylation is closely associated with transcriptional activation [110]. Recently, Diomede et al. [103] investigated if epigenetic modulations is involved in periodontitis by using human periodontal ligament stem cells (hPDLSCs) as an *in vitro* model. They found that *P. gingivalis* LPS significantly reduced DNA methylase DNMT1, while it markedly upregulated the level of histone acetyltransferase p300 and NF-kB. This demonstrated that *P. gingivalis* LPS markedly regulates genes involved in epigenetic mechanisms, which may result in induction of inflammation locally and systemically.

## Molecular inhibitors as possible therapy in Alzheimer's disease

The role of inflammation in AD is well established. Interestingly, resolvin E1 and lipoxin A4 resolved the inflammation in a murine model of AD [111]. This leads to the question whether complement mediated therapy should also be considered to reduce the inflammatory load in AD and if so, when and how? Indeed, AD and periodontitis have complement-TLR intercommunication mediated inflammation in common. The contribution from peripheral sources to inflammatory mediators has an early impact on priming of intracerebral glial cells. An ideal window to control the impact of peripheral inflammation from periodontitis on AD would therefore appear to be from the time of diagnosis of the oral disease. The clinical value of inhibiting all the three main pathways of complement activation was recently suggested in periodontitis [19]. This can be achieved by targeting the central component C3, which directly inhibits inflammation and indirectly counteracts dysbiosis.

As for *P. gingivalis*, Dominy et al. [17] proposed that potent and selective gingipain inhibitors (Kgp) could be valuable for treating *P. gingivalis* colonization of the AD brain. Using effective molecular inhibitors of gingipains at later stages of this neurodegenerative disease may be tried, but there are many causes of AD and multiple bacterial phylotypes discovered in demented brains.

## Concluding remarks

There is no generally accepted view on the pathogenesis of AD, which is considered a multifactorial disease. Recent research has shown that an impaired complement system plays an important role in the AD brain. Whether nature provided this early immune system to be protective, as suggested by the Aβ-AMP concept, or other forms of toxicity in old age is a subject open to debate. By affecting some of the gene defective proteins, *P. gingivalis* may amplify complement mediated inflammatory dysbiosis, but this must be proven.

Now that the GWAS has demonstrated the role of defective complement activation in AD development, this supports our working hypothesis that AD in some patients is mediated by the host’s inflammatory responses and justifies the rationale for novel interventions to improve lifestyle, behavior and regular dental care. However, there is no definite proof yet of a link in AD between defects in the complement cascade of innate immunity and *P. gingivalis*. This bacterium could rather emulate genes involved in epigenetic mechanisms by its LPS, which may result in induction of inflammation locally and systemically. Future research should try to establish a better foundation for the notion that there could be a genetic basis for *P. gingivalis* infection in AD.
